# Antiproliferative, Antioxidant, Chemopreventive and Antiangiogenic Potential of Chromatographic Fractions from *Anemonia sulcata* with and without Its Symbiont *Symbiodinium* in Colorectal Cancer Therapy

**DOI:** 10.3390/ijms241411249

**Published:** 2023-07-08

**Authors:** Mercedes Peña, Cristina Mesas, Gloria Perazzoli, Rosario Martínez, Jesús M. Porres, Kevin Doello, Jose Prados, Consolación Melguizo, Laura Cabeza

**Affiliations:** 1 Institute of Biopathology and Regenerative Medicine (IBIMER), Center of Biomedical Research (CIBM), University of Granada, 18100 Granada, Spain; mpenacontreras@ugr.es (M.P.); cristinam@correo.ugr.es (C.M.); gperazzoli@ugr.es (G.P.); kdoello@correo.ugr.es (K.D.); melguizo@ugr.es (C.M.); lautea@ugr.es (L.C.); 2 Department of Anatomy and Embryology, Faculty of Medicine, University of Granada, 18071 Granada, Spain; 3 Biosanitary Institute of Granada (ibs.GRANADA), SAS-University of Granada, 18014 Granada, Spain; 4 Department of Physiology, Institute of Nutrition and Food Technology (INyTA), Center of Biomedical Research (CIBM), University of Granada, 18100 Granada, Spain; rosariomz@ugr.es (R.M.); jmporres@ugr.es (J.M.P.); 5 Medical Oncology Service, Virgen de las Nieves Hospital, 18016 Granada, Spain

**Keywords:** *Anemonia sulcata*, *Symbiodinium*, fractionation, antioxidant activity, chemopreventive activity, antiangiogenic activity, antitumor activity, colorectal cancer

## Abstract

*Anemonia sulcata* may be a source of marine natural products (MNPs) due to the antioxidant and antitumor activity of its crude homogenates shown in vitro in colon cancer cells. A bioguided chromatographic fractionation assay of crude *Anemonia sulcata* homogenates with and without its symbiont *Symbiodinium* was performed to characterize their bioactive composition and further determine their biological potential for the management of colorectal cancer (CRC). The 20% fractions retained the in vitro antioxidant activity previously reported for homogenates. As such, activation of antioxidant and detoxifying enzymes was also evaluated. The 40% fractions showed the greatest antiproliferative activity in T84 cells, synergistic effects with 5-fluoruracil and oxaliplatin, overexpression of apoptosis-related proteins, cytotoxicity on tumorspheres, and antiangiogenic activity. The predominantly polar lipids and toxins tentatively identified in the 20% and 40% fractions could be related to their biological activity in colon cancer cells although further characterizations of the active fractions are necessary to isolate and purify the bioactive compounds.

## 1. Introduction

The oceans cover 70% of the Earth’s surface and contain a wide variety of organisms and species, resulting in enormous chemical diversity. Numerous marine natural products (MNPs) with biological activity and potential applications in the pharmaceutical, biomedical, and even nutraceutical fields have been described. At least 20 marine drugs are currently approved for clinical use and approximately 35 drugs are under clinical investigation, most of them used in antineoplastic therapy [1,2]. Among this biodiversity, invertebrates are one of the most important sources of new MNPs, with the Porifera and Cnidaria phyla being significant sources of cytotoxic and antitumor compounds [3,4].

One of the most common cnidarians of the Mediterranean coasts is the sea anemone *Anemonia sulcata (A. sulcata)* (Pennat, 1777), or *Anemonia viridis* (Forsskål, 1775). *A. sulcata* establishes a light-dependent symbiotic relationship with zooxanthellae (Smith, 1939), which are photosynthetic dinoflagellate microalgae of the genus *Symbiodinium* [5]. Subjecting these organisms to adverse conditions, such as lack of light, can interrupt this symbiotic relationship in a process called bleaching [6]. This anemone has stinging cells in its tentacles (cnidocytes), with nematocysts that release venom containing various active molecules, including potent toxins that affect voltage-dependent Na+ and K+ channels or protease inhibitors whose bioactive potential has been poorly studied [7]. Previous studies have shown relevant biological activities of *A. sulcata*, including anti-inflammatory activity [8] and the prevention of Alzheimer’s disease due to the neuroprotective role of the toxin BDS-I [9]. In relation to cancer treatment and prevention, *A. sulcata* has shown antiproliferative effects on cancer cells [8,10,11], antiangiogenic potential [12], and intrinsic antioxidant activity [13,14]. Moreover, our previous results showed higher antitumor and antioxidant activity with the treatment of crude *A. sulcata* homogenates subjected to the bleaching process, probably due to the metabolic stress generated [10].

Currently, colorectal cancer (CRC) remains the third most common tumor in incidence and the second leading cause of cancer death worldwide [15]. At the time of diagnosis, 20% of CRC patients have metastases and therefore a poor prognosis, as treatment options are limited. Pharmacological treatments in patients with advanced stages of the disease include chemotherapy with drugs including 5-fluorouracil (5FU), irinotecan, capecitabine, or oxaliplatin (OXA), in combinations such as FOLFOX or FOLFOXIRI, and monoclonal antibodies such as cetuximab or panitumumab (anti-EGFR). The antiangiogenic agents bevacizumab and aflibercept (anti-VEGF) or the recently developed regorafenib and TAS-102 are also used [16,17]. However, the non-specificity of treatments and the multidrug resistance (MDR) of tumor cells are limitations that affect the quality of life and prognosis of patients with CRC. One of the key factors in tumor resistance and recurrence is the existence of cancer stem cells (CSCs) within the heterogeneity of tumor cells with the capacity for self-renewal and differentiation; this makes them responsible for tumor recurrence [18]. Therefore, the development of new therapies and preventive strategies or approaches that improve existing treatments is necessary to improve the survival and quality of life of patients with CRC.

In response to these demands, the vast biodiversity of marine organisms may be a source of new bioactive compounds (cytarabine [19], eribulin [20], trabectedin [21], etc.) and/or enhancers of the existing treatments [22,23]. Currently, there is no clinical evidence of MNP synergy with chemotherapeutic drugs [24,25,26,27] and the studies related to this are mainly based on plant extracts [28,29,30,31,32,33]. Furthermore, few marine-derived drugs (bryostatin-1, panobinostat, plitidepsin, marizomib, and plinabulin) have been successfully evaluated in clinical trials in patients with cancer [34] and specifically in patients with CRC. However, some marine products (plitidepsin, plocabulin, spisulosin, trabectedin) have reached phase I clinical trials [35,36,37,38]. Thus, although plants are being widely studied, more research on marine organisms is needed. In the present work, crude homogenates from *A. Sulcata* with and without symbiont (W and W/O, respectively) obtained through a bleaching process were subjected to a bio-guided fractionation to elucidate their bioactive composition and further investigate their biological potential. Additional characterization of the most active fractions is necessary to isolate and purify the bioactive compounds for CRC management.

## 2. Results

### 2.1. Antiproliferative Activity in CRC Cultured Cells

**Figure 1:**
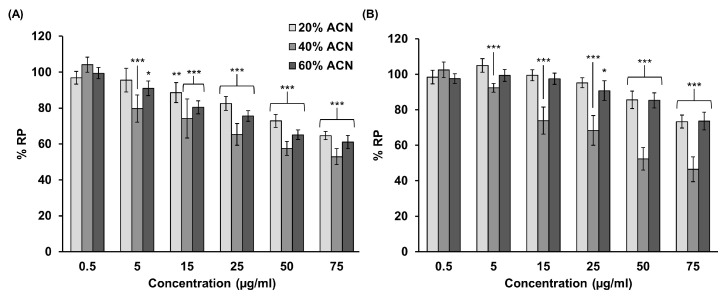
Antiproliferative activity of chromatographic fractions of crude homogenate from *A. sulcata*. T84 colorectal cancer cells were treated with three acetonitrile-eluted chromatographic fractions (20, 40, 60%) from (**A**) HOMG W and (**B**) W/O at a concentration range of 0.05 to 75 µg/mL for 72 h. Relative proliferation (%RP) was calculated as the mean ± standard deviation (SD) of 3 replicates from two different experiments measured by MTT colorimetric assay. Statistically significant differences compared to untreated cells were calculated: *p* value < 0.05 (*); <0.01 (**); <0.001 (***).

The antiproliferative activity of the three chromatographic fractions (20, 40, 60% acetonitrile), obtained during reverse phase fractionation of crude homogenates (HOMG) of *A. sulcata* with (W) and without (W/O), was tested in T84 cells at increasing concentrations for 72 h (Figure 1). Most of the chromatographic fractions from both crude homogenates reduced the relative proliferation (%RP) of T84 cells. The 40% fraction of both the crude HOMG W (53% RP) and HOMG W/O (47%RP) showed the highest cytotoxic effect, with IC_50_ (maximal inhibitory concentration) values of approximately 75 μg/mL.

### 2.2. Synergistic Effect Analysis

**Figure 2:**
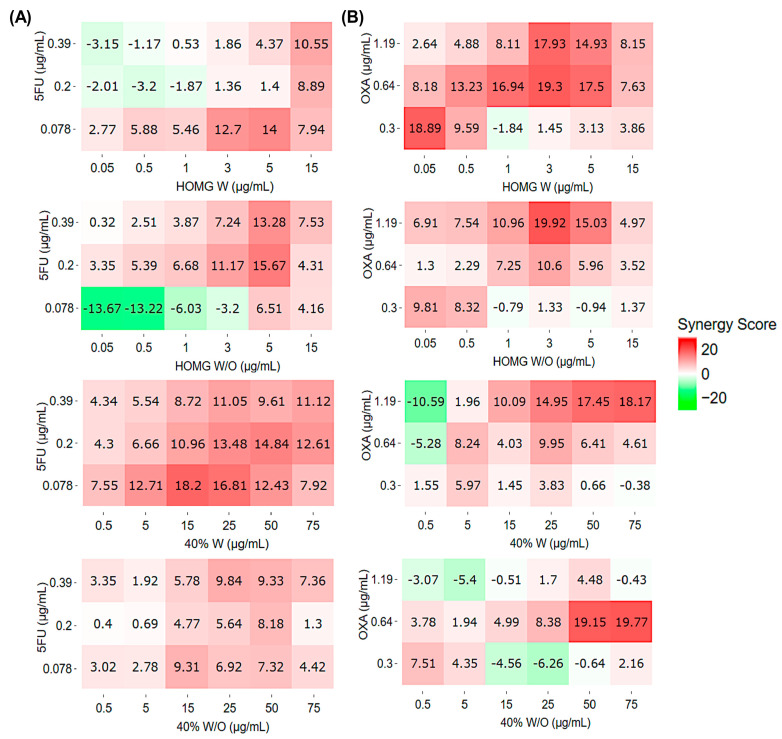
Heatmaps of T84 cells treated with crude homogenates or 40% fractions from *A. sulcata* in combination with (**A**) 5FU or (**B**) OXA. Different concentrations of HOMG W, W/O, or 40% W and 40% W/O were co-incubated with 5FU (0.08, 0.2 and 0.39 μg/mL, respectively) or OXA (0.3, 0.64, and 1.19 μg/mL, respectively) for 72 h. Synergy scores (HLA model) were represented in Heatmaps with SynergyFinder Plus, where areas of synergy (values >10 in red color), additive effect (from −10 to 10 in white color), and antagonism (<−10 in green color) were distinguished.

Synergy analysis using the HLA (highest single agent) model revealed a slight synergistic effect between HOMG W or W/O and 5FU (Figure 2A). Maximum scores were obtained with a combination of 5 μg/mL of HOMG W or W/O and 5FU at 0.078 (score: 14) or 0.2 μg/mL (score: 15.67), respectively. Furthermore, the 40% W fraction demonstrated the greatest synergistic effect with multiple combinations with HLA synergy scores > 10 and a maximum value of 18.2 (15 μg/mL of 40% W and 0.078 μg/mL of 5FU). However, 40% W/O co-incubation with 5FU resulted in an HLA synergy score between −10 and 10, suggesting an additive effect. On the other hand, in combination with oxaliplatin, both the homogenates and the fractions reached synergy scores > 10, with maximum scores of around 18–19 in all cases (Figure 2B). The combinations of HOMG W (0.05 and 3 μg/mL) with OXA (0.3 and 0.64 μg/mL, respectively) obtained the maximum scores: 18.89 and 19.3, respectively. Cotreatment of HOMG W/O (3 μg/mL) and OXA (1.19 μg/mL) reached a synergy score of 19.92. The highest concentrations of the 40% W and W/O fractions demonstrated the greatest synergistic value by the HLA method in combination with oxaliplatin (1.19 and 0.64 μg/mL, respectively), with maximum scores of 18.17 and 19.77, respectively. Analyses of the ZIP (zero interaction potency) (Figure S1), Loewe (Figure S2), and Bliss (Figure S3) methods yielded lower synergistic effects. While the Loewe method showed similar results to HLA, although less potent, the ZIP and Bliss methods suggested an antagonistic trend.

### 2.3. HCT116-Tumorspheres Viability Assay

**Figure 3:**
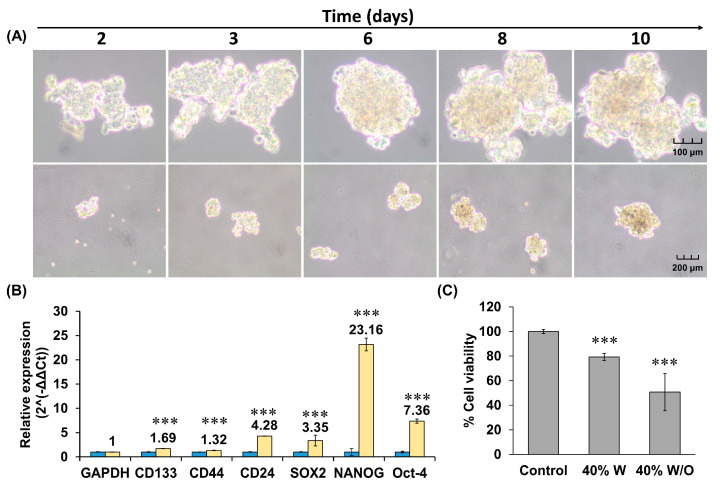
HCT116 tumorspheres formation, characterization, and treatment with 40% chromatographic fractions from *A. sulcata*. (**A**) Light microscopy images of tumorspheres after 10 days of induction. (**B**) Relative expression of CSC-related markers in HCT116 tumorspheres by RT-qPCR. (**C**) Viability assay of HCT116 tumorspheres treated with 40% W and W/O (400 μg/mL) using CCK8 assay. Results are expressed as % cell viability with respect to the total population of control cells. Statistically significant differences compared to untreated cells were calculated: *p* value < 0.001 (***).

After 10 days, HCT116 cells cultured with the serum-free medium, described previously, under non-attachment conditions formed tumorspheres (Figure 3A). These spheres were characterized by RT-qPCR and showed statistically significant overexpression of all cancer stem cell (CSC) markers (Figure 3B). Once the CSCs’ phenotype was confirmed, the tumorspheres were subjected to a concentration of 4xIC_50_, previously obtained in the HCT116 cell line, of the 40% W and 40% W/O chromatographic fractions (400 μg/mL) (Figure 3C). The 40% W and W/O fractions significantly affected the percentage of cell viability; the latter was the one that obtained the greatest cytotoxic effect, with a reduction up to 50.71% when compared to the control.

### 2.4. Protein Expression of Caspases 8 and 9, and PARP1

**Figure 4:**
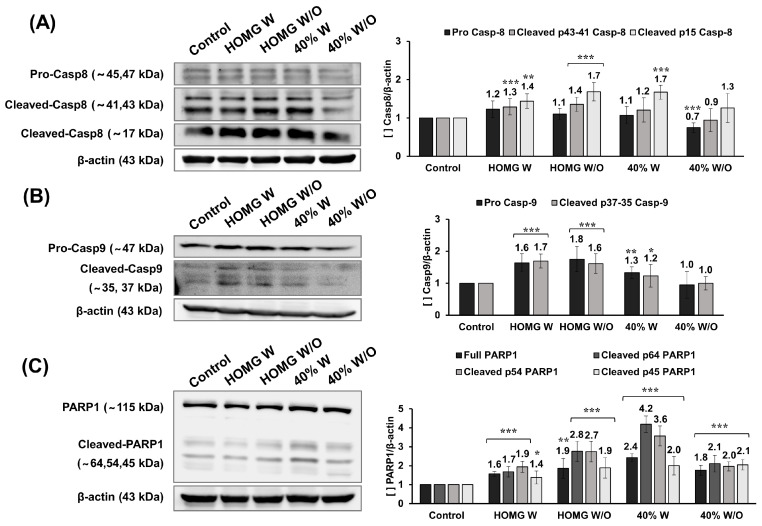
Western blot analysis of (**A**) caspase 8, (**B**) caspase 9, (**C**) PARP1 protein expression from cells treated with HOMG W and W/O, and 40% W and W/O. The bands obtained by chemiluminescence were analyzed using Quantity One 4.6.8 analytical software, and relative expression (Protein/β-actin) was calculated as the mean ± SD of three measurements from three Western Blot replicates. Statistically significant differences compared to untreated cells were calculated: *p* value < 0.05 (*); <0.01 (**); <0.001 (***).

Western Blot analysis showed a statistically significant increase in cleaved caspase 8 and 9 protein expression in cells treated with HOMG W and W/O when compared to control cells (Figure 4A,B). Higher levels of caspase 8 cleavage fragments (43–41 and 15 kDa) were observed in HOMG W (1.3 and 1.4-fold vs. control, respectively) and W/O (1.4 and 1.7-fold vs. control, respectively). Among the fractions, the 40% W fraction showed increased expression of cleaved caspase 8 (15 kDa). Moreover, the expression of procaspase 9 and cleaved caspase 9 was increased by HOMG W (1.6 and 1.7-fold vs. control, respectively) and W/O (1.8 and 1.6-fold vs. control, respectively), as well as 40% W fraction (1.3 and 1.2-fold vs. control, respectively). However, no alteration in protein expression was observed with 40% W/O treatment. In relation to PARP1, all treatments resulted in a statistically significant increase in the expression of the full-length protein and three cleavage fragments (64, 54, 45 kDa), reaching the highest expression level in p64 and p54 cleaved by HOMG W/O (2.8 and 2.7-fold, respectively vs. control) and 40% W (4.2 and 3.6-fold, respectively vs. control) (Figure 4C).

### 2.5. Antiangiogenic Activity

**Figure 5:**
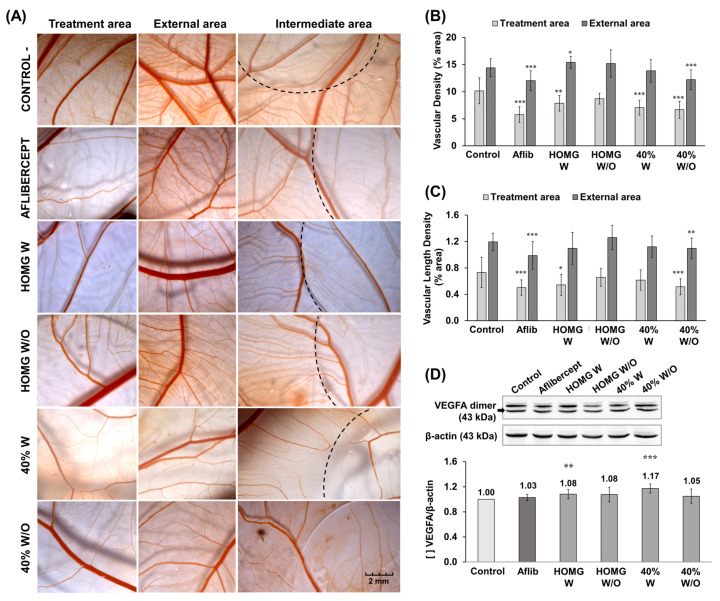
Study of the antiangiogenic potential of crude homogenates and 40% chromatographic fraction of *A. sulcata*. (**A**) Images of both treatment and external areas of CAM subjected to different conditions (15 μL/egg): negative control (saline solution), positive control (Aflibercept [Aflib], 10 mg/mL), HOMG W and W/O (0.5 mg/mL), and 40% W and W/O (0.5 mg/mL). Images were analyzed with the plugin “Vessel Analysis” from FIJI and the % area of (**B**) vascular density and (**C**) vascular length density were obtained for each condition. (**D**) Protein VEGFA expression from each condition was studied by Western Blot analysis. Bands obtained by chemiluminescence were analyzed using Quantity One analytical software, and relative expression (VEGFA/β-actin) was calculated as the mean ± SD of three measurements from three Western Blot replicates. Statistically significant differences compared to the negative control were calculated: *p* value < 0.05 (*); <0.01 (**); <0.001 (***).

The antiangiogenic potential of *A. sulcata* W and W/O homogenates and their respective 40% fractions was assessed by Chick Chorioallantoic Membrane (CAM) assay by administering the treatments on a round coverslip on the CAM surface. After 72 h of treatment, the images showed differences in vascular density between the outer and inner areas of the coverslip in all conditions (Figure 5A). The results of the CAM assay demonstrated antiangiogenic activity for HOMG W and W/O (0.5 mg/mL), with statistically significant differences observed in the HOMG W treatment (reduction of 2.3% area in vascular density vs. negative control). Moreover, both the 40% W and W/O fractions (0.5 mg/mL) exhibited a statistically significant decrease (3.08% and 3.49% area, respectively) in vascular density in the area of treatment when compared to the negative control (Figure 5B). The 40% W/O fraction also showed a significant 0.22% area reduction in vascular length density (Figure 5C). Western blot results revealed similar VEGFA (Vascular Endothelial Growth Factor) expression in all conditions, including the positive control. Therefore, no significant reduction in protein expression was observed in any of the treatments when compared to the non-treated cells (Figure 5D).

### 2.6. In Vitro Antioxidant Activity

**Figure 6:**
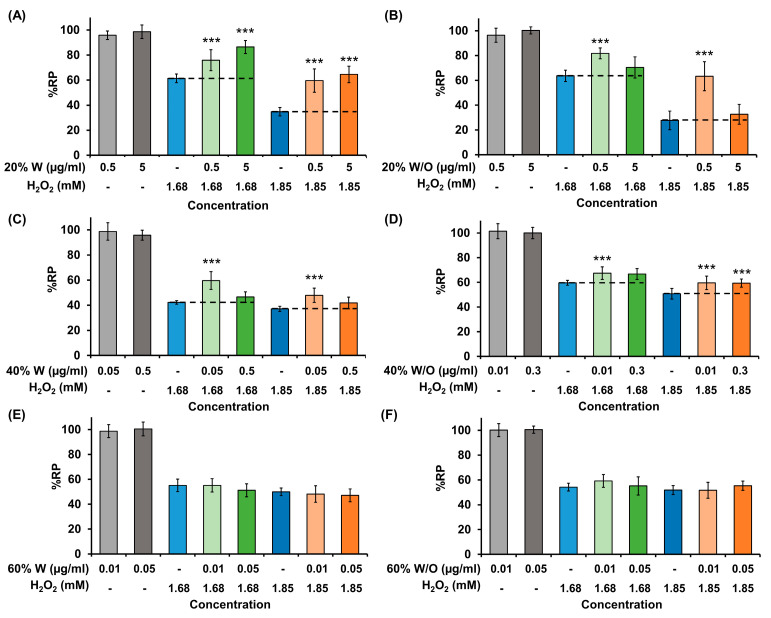
Antioxidant activity of chromatographic fractions of *A. sulcata* in vitro. HT29 colorectal cancer cells were subjected to a 24 h pretreatment with two different non-cytotoxic concentrations of each of the fractions from HOMG W and W/O: (**A**) 20% W; (**B**) 20% W/O; (**C**) 40% W; (**D**) 40% W/O; (**E**) 60% W; and (**F**) 60% W/O. After this, the medium was changed, and cells were incubated for 6 h with two doses of H_2_O_2_ (1.68 and 1.85 mM). Relative proliferation (%RP) was obtained by MTT assay. Data are represented as the mean ± SD of 8 replicates. Statistically significant differences compared to cells treated with H_2_O_2_ only were calculated: *p* value < 0.001 (***).

Non-cytotoxic concentrations of the 20% and 40% fractions W and W/O were able to increase the viability of cells treated with H_2_O_2_ (Figure 6). Both concentrations of the 20% fraction from HOMG W (0.5 and 5 μg/mL) increased cell viability by approximately 15–25% (1.68 mM H_2_O_2_) and 25–30% (1.85 mM H_2_O_2_), respectively. The highest protective effect was achieved with the 20% fraction from HOMG W/O (0.5 μg/mL), which increased the viability of HT29 cells treated with 1.85 mM H_2_O_2_ by 36%. A significant protective effect was also observed with the 40% fraction W (0.05 μg/mL concentration increased cell viability by 17.4% and 10.8% compared to cells treated with 1.68 and 1.85 mM H_2_O_2_, respectively) and W/O (both 0.01 and 0.03 μg/mL concentrations increased cell viability by 8.8% and 8.6% compared to cells treated with 1.85 mM H_2_O_2_, respectively. However, the protective effect was observed to a lesser extent in this latter example than the 20% fractions. On the other hand, the 60% W and W/O fractions did not increase the viability of cells treated with H_2_O_2_ at non-cytotoxic concentrations.

### 2.7. Determination of Antioxidant Enzyme Induction Capacity

**Figure 7:**
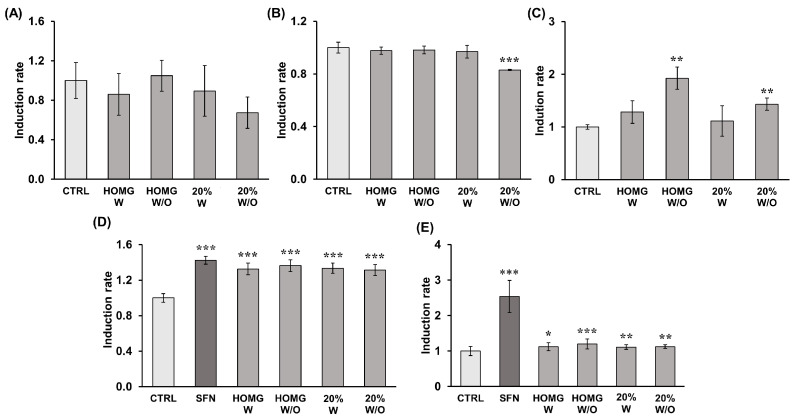
(**A**) Catalase (CAT), (**B**) Superoxide dismutase (SOD), (**C**) Glutathione peroxidase (GPX), (**D**) Glutathione S-Transferase (GST), and (**E**) NAD(P)H: quinone oxidoreductase (QR) activity induced by cytosolic fractions from *A. sulcata* with and without symbiont homogenates and their respective 20% ACN fraction. Cytosolic fractions were obtained from cell cultures treated with HOMG W and W/O (0.5 µg/mL) and 20% W and W/O fractions (5 µg/mL) for 48 h. GST and QR activity was measured at 340 and 600 nm, respectively, and UA/mg protein and induction rate (treated/control) were calculated. Cells treated with sulforaphane (SFN) (1.77 µg/mL) were used as the positive control. Statistically significant differences compared to untreated cells were calculated: *p* value < 0.05 (*); <0.01 (**); <0.001 (***).

The results of the antioxidant activity of catalase (CAT), superoxide dismutase (SOD), and glutathione peroxidase (GPX) enzymes are summarized in Table 1. Enzyme measurement data showed that all treatments with *A. sulcata* significantly increased the activity of the antioxidant enzyme GPX for HOMG W/O and 20% W/O (1.93 and 1.43-fold, respectively) when compared to the activity of untreated cells. CAT and SOD showed no significant differences between treatments and control (Figure 7).

### 2.8. Determination of Detoxifying Enzyme Induction Capacity

The results of measuring the activity of the detoxifying enzymes Glutathione S-transferase (GST) and NAD(P)H: quinone oxidoreductase (QR) of the cytosolic fractions obtained from cells treated with the crude homogenates of *A. sulcata* and their respective 40% fractions are shown in Table 1. Sulforaphane (SFN) (1.77 µg/mL), used as a positive control, showed a significant increase in enzyme activity when compared to the negative control in both assays (37.98 UA/mg increment in GST and 1489.3 UA/mg in QR). In the GST assay, HOMG W and W/O and the 40% W and W/O fractions showed similar activity to each other but with a significant improvement in activity (≈1.33-fold) when compared with the activity of untreated cells. QR enzyme activity was also statistically significantly affected by each of the *A. sulcata* treatments in a similar way. Specifically, HOMG W/O showed a greater increase (1.20-fold) than the rest of the treatments (1.1-fold) when compared to the QR activity of the control (Figure 7).

### 2.9. Chemical Characterization of Active Fractions

#### 2.9.1. Composition Analysis of the Active Fractions by HPLC-MS

**Figure 8:**
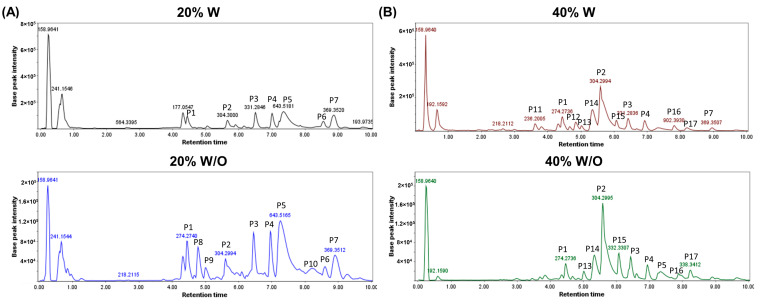
HPLC-MS chromatographic profiles of chromatographic fractions obtained from *A. sulcata* with and without symbiont homogenates. The most intense peaks of (**A**) 20% fraction W and W/O and (**B**) 40% W and W/O were highlighted and tentatively identified.

HPLC-MS analysis of the 20% W and W/O and 40% W and W/O fractions revealed similar chromatographic profiles, with several peaks in common (Figure 8). Table S1 shows the suggested molecular formulae and the corresponding compounds for each of the m/z values found in different peaks. A total of five compounds have been tentatively identified as sphingolipids, including sphingoids such as Sphinganine, phosphosphingolipids, and ceramides (Cer). Four of the total peaks were suggested to correspond to glycerophospholipids, including glycerophosphocholines (PC) and glycerophosphoethanolamines (PE). Glycerolipids of the monoradylglycerols class were also proposed for two compounds found. Other lipid molecules were tentatively identified as fatty acids (fatty amides and hydrocarbons) and sterols (brassicasterol and cholesterol). On the other hand, three of the m/z obtained were associated with tripeptides such as -His Lys Met-, -Arg Trp Ala-, and -Lys Gln Tyr-. Although the chromatograms obtained were similar, differences were found in the composition of the 20% and 40% fractions. The peaks from P11 to P17, which include the described tripeptides, appeared in higher proportion in the 40% W and W/O fractions. Additionally, P6, corresponding to Brassicasterol, suggested that the sterol lipid was identified with higher intensity in both the 20% W and W/O fractions when compared to the respective 40% fractions. Differences were also observed between fractions of anemone homogenates with and without symbiont, especially in the 20% fraction. Some glycerophospholipids, such as PE(20:5(5Z,8Z,11Z,14Z,17Z)/0:0), PC(14:1(9Z)/0:0), or PE(17:1(9Z)/0:0); phosphosphingolipids, similar to PE-Cer(d14:1(4E)/21:0), and ceramides Cer(d14:1/20:1) or Cer(d18:2/16:0), seemed to be present only in the 20% W/O fraction.

#### 2.9.2. Protein Identification by LC-MS/MS

The summarized results of the analysis of the protein composition by LC-MS/MS of the 20% and 40% active fractions of *A. sulcata* are shown in Table 2. A total of 48 proteins have been identified, of which 36 corresponded to toxins described in *A. sulcata* and/or *A. viridis*. These toxins have mainly been classified according to the following families: sodium voltage-gated (Nav) channel inhibitor toxins; potassium voltage-gated channel type 1 toxins (Kv1); potassium voltage-gated channel type 2 toxins (Kv2, Venom Kunitz-type family); and potassium voltage-gated channel type 3 toxins (Kv3, Blood depressing substance or BDS family). Some toxins of the Venom Kunitz-type family, such as AsKC1 (kalicludine-1), AsKC2 (kalicludine-2), AsKC3 (kalicludine-3), AsKC4, and AsKC9, together with BDS-13 and Avtx-4, were the only toxins identified in the four fractions analyzed. Most of the remaining toxins were present only in the 40% fractions, including the sodium channel inhibitors ATX-V (Delta-AITX-Avd1d), Toxin 2-4 (Delta-AITX-Avd1e 1), and neurotoxin 1-1 (Delta-AITX-Avd1h), toxins of the BDS family, including BDS-3, BDS-4, BDS-7, and BDS-10 (Table S2), or several type III potassium channel isotherms, among others. Kaliseptine toxin (Kappa-AITX-Avd6a) was the only toxin identified in the 20% fractions but not in the 40% fractions. Conversely, antioxidant enzymes such as catalase, copper/zinc superoxide dismutase (CuZnSODb), and glutathione peroxidase were found in both the 20% and 40% fractions W and W/O, except for glutathione peroxidase, which was not found in the 20% W fraction.

## 3. Discussion

Marine organisms are an important source for the discovery of new active compounds that may be used in cancer therapy. Strategies to manage CRC range from prevention to treatments administered alone or in combination with other drugs. Based on our previous results [10], we performed a bio-guided fractionation assay in the present work to characterize the bioactive potential of *A. sulcata* as a source of compounds with antitumor and/or antiangiogenic activity for CRC treatment and antioxidant and/or chemopreventive activity for its prevention. The cytotoxic activity of the fractions obtained was higher with the treatment of the 40%W and W/O fractions on T84 cells with IC_50_ ~ 75 ug/mL. The antiproliferative activities of 45% and 60% ACN fractions (50–200 μg/mL) from *A. viridis* have also been previously observed in tumor cell lines such as PC3, PLC/PRF/5, and A375 [11]. Furthermore, HCT116-derived tumorspheres with overexpression of typical colon CSC markers (CD133, CD44, CD24, SOX2 NANOG, and Oct-4) [39,40,41] showed a reduction in their viability after treatment with 40% W and W/O fractions of *A. sulcata* as is the case with numerous marine-derived compounds, such as fucoxanthinol or sphaerococcenol A [42,43,44,45], which are of great importance in the prevention of tumor self-regeneration and cell resistance to conventional drugs.

In combination with chemotherapeutic drugs commonly used to treat CRC, such as 5FU and OXA, 40% W and W/O exhibited synergistic effects as determined by the HLA method (40% W/O had an additive effect with a synergistic tendency). Natural compounds combined with conventional therapies may reduce resistance to treatment, increase cell sensitivity, and reduce the therapeutic dose, thus minimizing adverse drug effects [28,29,30,31,32,33,46]. A synergistic effect has even been described between OXA and docosahexaenoic acid (DHA), an omega-3 polyunsaturated fatty acid present in *A. sulcata*, according to our previous study of the fatty acid profile [10], through autophagy increment via the increase of endoplasmic reticulum stress and expression of Sestrin 2 [47].

Regarding the possible mechanism of cell death induced by *A. sulcata*, an increase in the expression of caspase 8 and 9 cleavage fragments was observed in cells treated with *A. sulcata* homogenates (W and W/O). The 40% W fraction exhibited a significant overexpression of caspase 8 (15 kDa) and 9 (37 and 35 kDa) cleavage fragments, but no increase was observed with the 40% W/O treatment. Our previous results suggested that the cytotoxic effect of *A. sulcata* homogenates could be related to apoptosis [10]. Activation of caspases 8 and 9 may be involved in both the extrinsic apoptosis pathway, mediated by death factors that bind to specific receptors, and the intrinsic or mitochondrial pathway [48]. Moreover, PARP1 expression improved in all treatments, being higher with the 40% W fraction treatment (64 and 54 kDa fragments) [49]. These could be associated with cell death mediated by cathepsins B, D, G, or granzyme B, which generate PARP1 fragments with a range of molecular weights (42–65 kDa) [50].

Regarding the tentative composition of the obtained fractions, HPLC-MS analysis revealed differences in the composition of the 20% and 40% fractions. In the 20% fraction, three possible tripeptides were tentatively identified, having demonstrated antitumor activity of some marine small peptides (<1000 Da) in previous studies [51,52,53]. In the 40% fractions, the HPLC analysis tentatively identified the presence of sphinganine (a sphingoid base similar to sphingosine) which induces apoptosis and G_2_/M cell cycle arrest in colon cancer cells [54]. Moreover, analysis of the protein composition of the 40% fractions by LC-MS/MS identified numerous toxins from *A. sulcata* and *A. viridis*, including neurotoxins that block sodium channels, potassium channel blockers such as kaliseptine, blood depressants (BDS), and Kunitz-type inhibitors of proteolytic enzymes (kalicludines). Furthermore, differences in the composition of toxins were observed between 20% and 40% chromatographic fractions. Neurotoxin 5, toxin 2-4, and BDS (3, 4, 10 and 13) were found in the 40% W and W/O fractions with high scores but not in the 20% fraction. These peptide toxins are ion channels blockers or modulators, whose expression and function in cancer cells, including CRC, have been previously studied [55]. The Nav inhibitory toxin family strongly affects Nav channels such as Nav1.5; these channels are upregulated in CRC cells and implicated in the metastatic potential of colon and ovarian cancers [56,57,58,59,60]. Dysregulation of potassium channels has also been implicated in tumor initiation and growth [61]. Toxins from sea anemones, including kaliseptine and kalicludine-1, -2, or -3, have been shown to inhibit Kv1.2 channels, which are overexpressed in various cancers [62]. BDS toxins block Kv3 channels, such as Kv3.1 and Kv3.4 subtypes, which play a role in cancer cell migration and invasion and have been detected in colonic crypt cells of mice with induced CRC and in the T84 cell line [63,64,65,66]. These findings suggest that the role of toxins in *A. sulcata* and their relation to antitumor potential should be further investigated.

Numerous MNPs have been described as angiogenesis inhibitors or blood vessel disruptors, and some of them are being evaluated in clinical trials [34]. Our results showed a decrease in vascular density by CAM assay without reducing VEGFA protein expression with HOMG W, 40% W, and 40% W/O treatments, similar to Aflibercept treatment. This antiangiogenic potential was not associated with the modulation of VEGFA expression in tumor cells but could be related to the VEGF pathway. Our proteomic analysis reported the presence of several BDS toxins in the 40% W and 40% W/O fractions, such as BDS-3, -4, -5, -7, -10, and -13, alongside the Arginine Glycin Aspartate (RGD) motif, which can interact with integrins and bind VEGF, resulting in antiangiogenic activity (Table S2) [12,67]. There are eight integrin dimers (αvβ1, αvβ3, αvβ5, αvβ6, αvβ8, α5β1, α8β1, and αIIbβ3) that recognize the RGD motif within proteins of the extracellular milieu; its blockade leads to strong inhibition of angiogenesis and subsequent apoptosis of endothelial and tumor cells [68].

Prevention has been associated with the administration of antioxidant compounds. These can inhibit the oxidation process by scavenging cell-toxic free radicals [69]. The LC-MS/MS analysis confirmed the presence of antioxidant enzymes such as catalase, copper/zinc superoxide dismutase (CuZnSODb), and glutathione peroxidase in both the 20% and 40% fractions (W and W/O symbiont). These enzymes are known to be present in sea anemones as endogenous scavenging systems for ROS [70]. However, the activity of catalase and superoxide dismutase in cultured cells was not significantly altered by *A. sulcata*, while glutathione peroxidase activity increased in all treatments, with statistically significant differences only in HOMG W/O and 20% W/O. This suggests that the antioxidant activity described in vitro could be mediated by the activation of the GPX enzyme by the fractions. The differences in the enzymatic activity between W and W/O could be due to the bleaching process of *A. sulcata* W/O, being subjected to greater stress and therefore a greater production of detoxifying enzymes [14]. The chemopreventive potential of *A. sulcata* was also investigated by measuring the activity of detoxifying enzymes, such as GST and QR, with increased enzymatic activities observed in both homogenates and 20% fractions when compared to untreated cells. This can be of great importance since natural marine compounds, especially those found in edible marine organisms such as *A. sulcata*, with great potential as chemopreventive agents, can be incorporated into the daily diet [71].

Characterization of the active fractions (20% and 40% W and W/O) by HPLC-MS suggested the presence of mostly polar lipids (PLs), including glycerolipids, glycerophospholipids (PC and PE), and sphingolipids (sphinganine and Cer) [6]. These PLs are important components of nematocysts and symbiotic dinoflagellates associated with the anemone [72,73], and could be associated with the observed antioxidant activity [74,75]. The presence of brassicasterol and cholesterol is suggested, particularly in the 20% fractions, since they are sterols previously described in this type of anemone [6]. Furthermore, previous experimental works have reported the antioxidant potential of marine sterols such as fucosterol to protect of oxidative damage [76]. In the 20% W/O fraction from *A. sulcata*, the presence of certain glycerophospholipids, phosphosphingolipids, and ceramides was observed, but not in 20% W. This could be due to the lack of light for the anemone W/O that leads to expanded lipid reserves rather than increasing structural growth [6,77].

## 4. Materials and Methods

### 4.1. Chemicals and Reagents

Chemicals including 1-chloro-2,4-dinitrobenzene (CDNB), 2,3-bis(2-methoxy-4-nitro-5-sulphophenyl)-2H-tetrazolium-5-carboxanilide (XTT), 2,6-dichloroindophenol (2,6-DCIP, sodium salt hydrate), 3-(4,5-Dimethylthiazol-2-yl)-2,5-Diphenyltetrazolium Bromide (MTT), 5-Fluorouracil (5-FU), acetonitrile (can), Bradford Reagent, cumene hydroperoxide, dimethyl sulfoxide (DMSO), flavin adenine dinucleotide disodium (FAD, salt hydrated), glutathione (GSH, reduced form), hydrogen peroxide (H_2_O_2_) solution, β-nicotinamide adenine dinucleotide (NAD, reduced disodium salt hydrated), β -nicotinamide adenine dinucleotide phosphate (NADPH), phosphate buffered saline (PBS), sodium chloride, sodium phosphate (NaH_2_PO_4_), sulforaphane (SFN), 0.1% aqueous trifluoroacetic acid (TFA) solution, and Trizma^®^ base were purchased from Sigma-Aldrich (Madrid, Spain). Absolute ethanol, di-sodium hydrogen phosphate anhydrous (Na_2_HPO_4_), and hydrochloric acid (HCl) were obtained from Panreac AppliChem (Barcelona, Spain). The Cell Counting Kit-8 (CCK-8) was purchased from Dojindo Laboratories (Munich, Germany).

### 4.2. Cell Culture

Human colorectal cancer cells (T84, HT-29 and HCT-116) were obtained from the American Type Culture Collection (ATCC). Cells were cultured in Dulbecco’s Modified Eagle’s Medium (DMEM) (Sigma-Aldrich) supplemented with 10% heat-inactivated fetal bovine serum (FBS) (Gibco, Madrid, Spain) and 1% of an antibiotic mixture (penicillin = 10.000 U/mL and streptomycin = 10 mg/mL) (Sigma-Aldrich). Cultures were maintained in an incubator with a humidified atmosphere at 37 °C and 5% CO_2_.

### 4.3. Animal Collection and Crude Homogenate Preparation

The *Anemonia sulcata* specimens with (W) and without (W/O) their symbiont microalgal *Symbiodinium*, inactivated by the bleaching process, were supplied by iMare Natural S.L (Granada, Spain). Three frozen anemones W and W/O symbionts were thawed on ice, washed with distilled water, weighed (≈39.94 g/W and ≈30.9 g/W/O), then homogenized in phosphate buffer (50 mM pH 7.2) using a mechanic homogenizer (IKA T10 Basic ULTRA-TURRAX9). The samples were then sonicated (1 min, 50% power, and cycles of 30 s) on ice and centrifuged (12,000 rpm, 15 min, 4 °C). The supernatants were collected, lyophilized, and stored at −80 °C [10].

### 4.4. Chromatographic Fractionation of Crude Homogenate from A. sulcata

Lyophilized crude homogenates from anemones (HOMG) W and W/O symbiont were fractionated using a Sep-Pak C18 Plus Short Cartridge (Waters, Milford, MA, USA) according to the modified protocol of Bulati et al. (2016) [11]. Briefly, C18 Cartridges were first preconditioned with (i) 100% methanol, (ii) double distilled water (ddW), and (iii) 0.1% aqueous trifluoroacetic acid (TFA) solution. Subsequently, the lyophilized samples were resuspended to a final concentration of 10 mg/mL in Trizma/NaCl buffer (10 mM/20 mM, pH 7.2) and slowly loaded onto the C18 cartridges. After loading the samples, the C18 Cartridges were washed with Trizma/NaCl buffer (10 mM/20 mM and pH 7.2) and the samples were eluted with a 20%, 40%, and 60% ACN gradient prepared in 0.1% TFA. The fractions obtained were collected separately and, after evaporation of the ACN, they were frozen at −80 °C, lyophilized, and resuspended in water for in vitro testing and chemical characterization.

### 4.5. Cell Viability Assay

T84 human CRC cell lines (5 × 10^3^) were seeded in 48-well plates in 300 μL of DMEM supplemented with 10% FBS and 1% antibiotics. After 24 h, chromatographic fractions from *A. sulcata* crude homogenates W and W/O symbiont were added at concentrations ranging from 0.5 to 75 μg/mL for 72 h. Untreated cells were the negative control. Following incubation, 30 μL of MTT (3-(4,5-Dimethylthiazol-2-yl)-2,5-Diphenyltetrazolium bromide) was added to each well for 3 h at 37 °C and 5% CO_2_. The medium was then removed, and the formazan crystals in each well were dissolved with a mixture of 200 μL of dimethyl sulfoxide (DMSO) and 25 μL of Sorensen’s glycine buffer (0.1 M glycine, 0.1 M NaCl, pH 10.5 with 0.1 NaOH). The optical density (OD) of samples was measured at 570 nm and 690 nm using a BioTek 800 TS absorbance reader (Agilent, Santa Clara, CA, USA). The relative proliferation (%RP) of the cells was calculated with the following formula:

$$\%RP=\frac{Treated\;cells\;OD}{Control\;OD}\;\times \;100$$

### 4.6. Synergistic Effect Analysis

T84 cancer cells were seeded in 48-well plates in 300 μL of supplemented DMEM. After 24 h, different concentrations of the crude homogenates W and W/O symbiont (0.05–15 μg/mL) and the chromatographic fraction of 40% ACN W and W/O (0.5–75 μg/mL) were co-incubated with the chemotherapeutic drugs 5-fluoruracile (5FU) (0.08, 0.2 and 0.39 μg/mL) or oxaliplatin (OXA) (0.3, 0.64 and 1.19 μg/mL) for 72 h. Expected drug combination responses were calculated based on HLA (highest single agent), LOEWE (Loewe additivity), BLISS (Bliss independence), and ZIP (zero interaction potency) reference models with SynergyFinder Plus (Helsinki, Finland) [78]. Full baseline correction was applied to data. Synergy scores were calculated as deviations between observed and expected responses and plotted on heat maps defining synergy (>10, in red color), additive effect (from −10 to 10, in white color), and antagonism (<−10, in green color) of the combined drugs.

### 4.7. Sphere Formation, Characterization, and Viability Assays

#### 4.7.1. Sphere Formation Assay

HCT116 cell tumorspheres were formed following the modified protocol of Feng et al. (2012) [79]. Briefly, 1 mL of 1% agarose (*w/v*) was added to 6-well plates. Once the agarose had gelled, HCT116 cells (2 × 10^3^) were grown in 2 mL of DMEM-F12 supplemented with 10% BSA (Sigma-Aldrich), 2% B27 supplement (Thermo Fisher Scientific, Waltham, MA, USA), 20 ng/mL of EGF and b-FGF, and 1% antibiotic mixture (Sigma-Aldrich). Tumorspheres were obtained after a minimum period of 10 days, with medium replacement every 5 days. Images were taken with an Olympus CKX41 inverted light microscope (Olympus Corporation, Tokyo, Japan) and tumorspheres were collected from plates for RNA extraction in order to test the expression of genes associated with the CSC phenotype by real time PCR and a viability assay.

#### 4.7.2. Real Time Quantitative PCR (qPCR)

HCT116 tumorspheres were characterized by evaluating the expression of markers associated with the CSC phenotype by real-time PCR. Adherent HCT116 cells and HCT116 tumorspheres in suspension were harvested, centrifuged, and resuspended in TRI Reagent^®^ (Sigma Aldrich, St. Louis, MI, USA). Total RNA was purified with RNeasy Micro Kit (Qiagen Iberia, S.L., Barcelona, Spain). Reverse transcription (RT) was performed with 2 × 1.5 μg RNA using Reverse Transcription System (Promega Biotech Iberica S.L., Madrid, Spain) following the manufacturer’s instructions. The RT-qPCR was conducted using primers for CD24, CD44, CD133, NANOG, SOX2, Oct-4, and glyceraldehyde-3-phosphate dehydrogenase (GAPDH) (endogenous control) genes. cDNA was amplified in a 96-well plate using StepOnePlus™ Real-Time PCR System (Applied Biosystems, Waltham, MA, USA, Thermo Fisher Scientific). The total reaction volume (20 μL) contained 60 ng cDNA, 1x TB Green Premix Ex Taq II and 1x ROX Reference Dye (Takara Bio Europe, Saint-Germain-en-Laye, France). The qPCR primers and annealing temperatures (Tm) used are listed in Table S3. qPCR was run at standard conditions and Ct values for all samples were determined automatically with the default settings in the StepOne Software V2.0 (Applied Biosystems, Thermo Fisher Scientific). Gene expression data were normalized using GAPDH and relative levels of gene expression were calculated using the 2^−∆∆Ct^ method.

#### 4.7.3. HCT116-Tumorspheres Viability Assay

After 10 days of induction, the tumorspheres were individualized with trypsin-EDTA solution (Sigma Aldrich) and seeded in 6-well plates (3500 cells in 2 mL/well). Following a 4 day incubation to reform the tumorspheres, cells were treated 40% ACN chromatographic fractions (400 μg/mL) for 72 h. Cell viability was assessed using the Cell Counting Kit-8 (CCK8) assay. These concentrations corresponded to 4 × IC_50_ (maximal inhibitory concentration) of treatments in HCT116 cell line, as calculated in previous experiments. 

### 4.8. Angiogenesis Assay—Chick Chorioallantoic Membrane (CAM) Assay

Fertilized eggs were obtained from a certified poultry farm. The experiment began on day 0, with the eggs cleaned with 70% ethanol and then incubated at 37.5 °C in a humidified atmosphere. After 3 days, 2 mL of albumen was removed from the apex of each egg and the natural air sac was pierced through a small hole at the blunt end. On the side of the eggshell, between these two holes, a 1.5 cm^2^ hole was made and covered with adhesive tape before the eggs were returned to the incubator in a horizontal position. On day 7, viable eggs were randomly divided in 6 groups (n = 10) and treated with 15 μL of the different treatments placed on a 13 mm round coverslip over the CAM with saline solution (negative control), 10 mg/mL Zaltrap^®^—Aflibercept (Sanofi, Paris, France) (positive control), 0.5 mg/mL HOMG W and W/O, and 0.5 mg/mL 40% fraction from both HOMG W and W/O. After 72 h, the CAM area was examined and photographed with a white background under the CAM with Motic SMZ-171 stereo microscope (Motic, Barcelona, Spain) and the images were analyzed with the FIJI “Vessel Analysis” plugin.

### 4.9. Western Blot Analysis (VEGFA, Caspase 8 and 9, PARP1 Expression)

T84 cells were seeded in T75 flasks (7.5 × 10^5^ cells in 8 mL of supplemented DMEM) and exposed to IC_50_ concentrations of crude HOMG W and W/O symbiont and their respective 40% fractions for 24 h (study of caspases and PARP1) or 72 h (study of VEGFA). Aflibercept (100 μg/mL) was used as a control antiangiogenic drug for the analysis of VEGFA expression. Cells were then detached, centrifuged, and total proteins were extracted using the Radio-Immunoprecipitation Assay (RIPA) lysis buffer (Thermo Fisher Scientific). Protein concentrations in the samples were determined by the Bradford assay. For electrophoresis, 40 μg of protein was denatured by heating at 95 °C for 5 min and separated on a 10% SDS-PAGE gel using a Mini Protean II cell (Bio-Rad, Hercules, CA). Then, the proteins were transferred from acrylamide gel to a nitrocellulose membrane with a 45 μm pore size (200 V at room temperature for 1.5 h) (Millipore). The protein-containing membrane was treated with blocking solutions (Phosphate-Buffered Saline (PBS)—0.1% Tween-20 + 7% (*w/v*) milk powder for VEGFA; Tris Buffered Saline (TBS)—0.1% Tween-20 + 5% (*w/v*) milk powder for PARP1, Caspase 9, and TBS—0.1% Tween-20 + 5% (*w/v*) BSA for Caspase 8), for 1 h, washed three times with PBS or TBS −0.1% Tween-20, and then incubated at 4 °C overnight with the primary antibodies: VEGFA (Rabbit anti-VEGFA antibody, ab46154, abcam, Cambridge, UK; 1:1000 dilution); Caspase 8 (Mouse anti-Caspase 8 antibody, MA1-41280, Invitrogen, Thermo Fisher Scientific; 1:1000 dilution), Caspase 9 (Mouse anti-Caspase 9 antibody, 9508, Cell Signaling Technology, MA, USA; 1:500 dilution), and PARP1 (Rabbit anti-PARP1 antibody, ab32138, abcam; 1:1000 dilution). The next day, after three washes, the membrane was incubated for 1 h at room temperature with the peroxidase-conjugated secondary antibody (anti-mouse IgGκ BP-HRP, sc-516102, Santa Cruz Biotechnology, CA, USA; 1:5000 dilution for caspase 8 and 9; and anti-rabbit IgG-HRP, sc-2357, Santa Cruz Biotechnology; 1:5000 dilution for VEGFA and PARP1). β-actin expression (Mouse anti-β-actin-peroxidase antibody, A3854, Sigma Aldrich; 1:25,000 dilution) was used as an internal control. Signals were detected by an ECL^TM^ Western blot detection reagent (Enhanced Chemiluminescence; Bonnus, Amersham, Little Chalfont, UK) and the bands obtained on the gels were analyzed with Quantity One 4.6.8 analytical software (Bio-Rad, Hercules, CA, USA).

### 4.10. Determination of In Vitro Antioxidant Activity

In vitro antioxidant activity of the chromatographic fractions of crude homogenates W and W/O symbiont from *A. sulcata* was measured in the human CRC cell line HT29 subjected to oxidative stress with hydrogen peroxide (H_2_O_2_) according to the protocol of Fuel et al. (2021) [80]. Briefly, HT29 cells were cultured in 96-well plates (2.5 × 10^4^ cells/well) in 150 μL of DMEM with 10% FBS and 1% antibiotics. The following day, the supplemented DMEM was replaced with serum-free DMEM and non-toxic concentrations of the treatments (0.01 and 5 μg/mL) were added after 24 h. The next day, the medium was discarded, and cells were treated with H_2_O_2_ (1.68 and 1.85 mM) for 6 h. Thereafter, the medium was replaced, and the next day an MTT assay was carried out as previously described.

### 4.11. Determination of Antioxidant and Detoxifying Enzymes Induction Capacity

To determine the antioxidant and detoxifying enzyme induction potential of anemone homogenates and fractions, cells were treated, the cytosolic fraction was purified, and the activity of antioxidant enzymes catalase (CAT), glutathione peroxidase (GPX), and superoxide dismutase (SOD) and detoxifying enzymes glutathione S-transferase (GST) and NAD(P)H: quinone oxidoreductase (QR) was measured following the modified protocol of Martínez et al. (2022) [81] and Fuel et al. (2021) [80], as detailed below.

#### 4.11.1. Treatment and Purification of the Cytosolic Fraction

HT29 colon cells were seeded in T25 flasks at a density of 1.5 × 10^6^ cells/well in 5 mL of supplemented DMEM. The following day, cells were exposed to non-cytotoxic doses of crude HOMG W and W/O (0.5 µg/mL) and their respective 20% fractions (5 µg/mL) for 48 h. Sulforaphane (1.77 µg/mL) was used as a positive control. After treatment, cells were trypsinized, washed twice with PBS, resuspended in 500 μL of 25 mM Tris-HCl (pH 6.4) for detoxifying enzymes or 50 mM phosphate buffer (pH 7.8) for antioxidant enzymes, and sonicated for 10 s at 40% frequency on ice. Subsequently, the cells were centrifuged at 10,000× *g*, 4 °C for 5 min, and the supernatant, that contains the cytosolic fraction, was used to determine the enzymatic activities. Protein concentration in the cytosolic fractions was established by Bradford assay.

#### 4.11.2. Antioxidant Enzyme Assays

Total superoxide dismutase (SOD) activity was measured as described by Ukeda et al. [82], with enzyme units defined as the amount required to inhibit 50% of the reduction of 2,3-bis(2-methoxy-4-nitro-5-sulphophenyl)-2H-tetrazolium-5-carboxanilide (XTT). Catalase (CAT) activity, expressed as enzyme units, was calculated by measuring H_2_O_2_ quenching in the presence of the cell cytosolic fraction at 240 nm, as described by Cohen et al. (1996) according to the formula: U = ln(Abs_1_/Abs_2_)/(t_2_ − t_1_), where “ln” is the natural logarithm, “Abs_1_” and “Abs_2_” are the absorbances observed at the two selected times (“t_1_” and “t_2_”) [83]. Total glutathione peroxidase (GPX) activity was determined by NADPH oxidation assay using cumene hydroperoxide as the substrate and defining the enzyme unit as nmol of NADPH oxidized per min [84].

#### 4.11.3. Detoxifying Enzyme Assays

Total glutathione S-transferase (GST) enzyme activity was measured by the colorimetric increase produced by the GST-catalyzed reaction of reduced glutathione (GSH) with the GST substrate, 1-chloro-2,4- dinitrobenzene (CDNB) (molar extinction 0.0096 μmol^−1^ cm^−1^). The reaction mix, containing 870 μL of 100 mM phosphate buffer (pH 6.5), 20 μL of 50 mM CDNB, and 10 μL of 100 mM GSH, was incubated at 30 °C for 5 min. Then, 100 μL of cytosolic supernatant of each sample was added to a quartz cuvette containing 900 μL of the reaction mixture, and the absorbance was measured at 340 nm every minute for 5 min in a UV-Vis Spectrophotometer UV-1900i (Shimadzu, Duisburg, Germany). The GST activity was calculated as the increase in absorbance/min/mg of total protein and compared with untreated cells.

The total NAD(P)H: quinone oxidoreductase (QR) enzyme activity was measured by the colorimetric reduction of 2.6-dichloroindophenol (DCIP) (molar extinction 0.0205 μmol^−1^/cm^−1^) by QR. The reaction mix, containing 881.5 μL of 25 mM Tris-HCl (pH 7.4), 60 μL of BSA (1 mg/mL), 2.5 μL of Tween (20%), 5 μL of 1 mM FAD, 10 μL of 20 mM NADH, and 16 μL of 5 mM DCIP, was incubated at 30 °C for 5 min. Then, 25 μL of each diluted sample (cytosolic supernatant) was added to a plastic cuvette containing 975 μL of the reaction mixture, and the absorbance at 600 nm was measured every minute for 5 min. QR activity was calculated as the decrease in absorbance/min/mg of total protein and compared with untreated cells.

### 4.12. Chemical characterization of Active Fractions

#### 4.12.1. HPLC-MS Analysis

Active chromatographic fractions were analyzed on an Agilent 1200 Rapid Resolution HPLC connected to a Bruker maXis mass spectrometer at MEDINA Foundation (Granada, Spain). A volume of 2 μL of the sample was injected into a Zorbax SB-C8 column (2.1 × 30 mm, 3.5 μm particle size) for the separation, using a flow rate of 0.3 mL/min with gradient from 90 to 0% A and from 10 to 100% B in 8 min (solvent A water:ACN 90:10 and solvent B water:ACN 10:90, both at 13 mM ammonium formate and 0.01% TFA). The mass spectrometer was set to positive ESI (ElectroSpray Ionization) mode, with the following parameters selected: 4 kV capillary voltage, 11 L/min at 200 °C for drying gas flow, and nebulizer pressure of 2.8 bar. Each of the chromatographic runs was processed using Bruker’s in-house algorithm for component extraction, and the most intense peaks in both positive TIC and absorbance at 210 nm were considered for the interpretation of exact masses and molecular formula. The combination of retention time and exact mass was used as the search criteria in the high-resolution mass spectrometry database of the MEDINA Foundation. For compounds with no matches in the above database, a search for the exact mass/molecular formula was performed in Chapman and Hall’s Dictionary of Natural Products.

#### 4.12.2. Protein Identification by LC-MS/MS (nLC-IT)

The protein concentration of active chromatographic fractions was determined by the Bradford assay. Sample proteins were isolated by loading 20 μg of protein, previously denatured by heating at 95 °C for 5 min, into a 20% SDS-PAGE gel using a Mini Protean II cell (Bio-Rad). Proteomics analyses were carried out by the Proteomics Unit from the Institute of Parasitology and Biomedicine “López-Neyra”, CSIC (Granada, Spain). First, the samples were digested using a manual digestion protocol. The protocol included reduction with dithiothreitol (DTT), derivatization with Iodoacetamide (IAM), and subsequent digestion with Trypsin (E:S 1:40) for 18 h at 30 °C. The resulting peptides were extracted from the gel with TFA 0.2% and 30% ACN for 1 h. The eluates were dried in Speed-Vac and stored at −20 °C. For LC-MS/MS analysis, the samples were resuspended in 0.2% formic acid (FA), 3% ACN. They were analyzed by nLC (easy nanoLC, Thermo) coupled to an ion trap mass spectrometer (Amazon Speed ETD, Bruker, Billerica, MA, USA) equipped with a Captive source. Chromatographic separation was performed on a C18 column (75 μm × 15 cm, 3 μm, 100 A) using a flow rate of 300 nL/min with a gradient from 5 to 30% B in 120 min (buffer A: 0.1% FA in water; buffer B: 0.1% FA in ACN). Three chromatographic runs per sample were performed. Mass analysis was performed in the range of 390–1400 (*m*/*z*). Ten precursors per cycle were selected for fragmentation, establishing a dynamic exclusion of 0.5 min. Protein identification was carried out using ProteinScape (Bruker) and MASCOT (Matrix Science, London, UK) as search engines. The search was performed in the SwissProt database with a filter for *Anemonia* and in the *Anemonia* protein database. *Anemonia* protein database (ID 6107) downloaded from NCBI (587 entries), considering carbamidomethylation of Cysteine as a fixed modification, and oxidation of Methionine as a variable modification.

### 4.13. Statistical Analysis

Results were expressed as the mean ± standard deviation (SD) of the replicates. Statistical analysis was performed using Student’s *t*-tests and one-way ANOVA with the Statistical Package for the Social Sciences (SPSS) v28.0.1 software. Data with a *p*-value < 0.05 were considered statistically significant.

## 5. Conclusions

This study highlights the diverse range of bioactive activities associated with the homogenates and their respective chromatographic fractions eluted with 20% and 40% acetonitrile derived from *A. sulcata* with (W) and without (W/O) symbiont. The 40% fractions maintained the antiproliferative activity previously observed in the crude homogenates, and exhibited synergistic antiproliferative effects with the CRC chemotherapeutic drugs 5FU and OXA; induction of caspase 8 and 9 overexpression, and PARP1; cytotoxic effects on colon cancer cell tumorspheres; and antiangiogenic activity. Moreover, both fractions retained the antioxidant capacity previously described in the homogenates, with the 20% fractions showing the highest antioxidant activity, potentially mediated by GPX activation. This fraction also demonstrated chemopreventive capacity by exhibiting higher activity of GST and QR enzymes when compared to untreated cells. Additionally, HPLC-MS analysis of the composition of the 20% and 40% fractions suggested the presence of polar lipids such as sphingolipids, glycerophospholipids, and glycerolipids, which could be associated with antioxidant or antitumor activities. Proteomic analysis identified toxins widely described in this organism that could be interesting candidates to evaluate their cytotoxic and antiangiogenic potential. Overall, the anemone *A. sulcata* has great potential as a source of pharmacologically active molecules applicable to the prevention and treatment of CRC. However, further research is needed to confirm the presence and involvement of the suggested bioactive compounds in the observed biological activities.

## Figures and Tables

**Table 1:** Antioxidant and detoxifying enzyme activity. Results are represented as mean ± SD of 3 measurements.

Antioxidant Activity						
	* CAT Activity		* SOD Activity		* GPX Activity	
	UA/mg protein	Induction Rate	UA/mg protein	Induction Rate	nmol NADPH/min/mg protein	Induction Rate
Control	1.39 ± 0.25	1.00 ± 0.18	3.43 ± 0.14	1.00 ± 0.04	4.68 ± 0.19	1.00 ± 0.04
Homg W	1.19 ± 0.29	0.86 ± 0.21	3.36 ± 0.09	0.98 ± 0.03	6.00 ± 1.00	1.28 ± 0.21
Homg W/O	1.45 ± 0.22	1.05 ± 0.16	3.37 ± 0.10	0.98 ± 0.03	9.01 ± 0.99	1.93 ± 0.21
20% W	1.24 ± 0.36	0.89 ± 0.26	3.33 ± 0.16	0.97 ± 0.05	5.21 ± 1.35	1.11 ± 0.29
20% W/O	0.93 ± 0.22	0.67 ± 0.16	2.85 ± 0.01	0.83 ± 0.00	6.70 ± 0.54	1.43 ± 0.12
**Detoxifying Capacity**						
	* GST activity			* QR activity		
	UA/mg protein		Induction Rate	UA/mg protein		Induction Rate
Control	89.65 ± 4.40		1 ± 0.049	967.58 ± 122.52		1.00 ± 0.126
Sulforaphane	127.63 ± 3.88		1.42 ± 0.043	2456.88 ± 435.17		2.54 ± 0.450
Homg W	118.92 ± 6.04		1.33 ± 0.067	1082.25 ± 108.94		1.12 ± 0.113
Homg W/O	122.24 ± 5.95		1.36 ± 0.066	1162.42 ± 136.91		1.20 ± 0.141
20% W	119.70 ± 5.13		1.34 ± 0.057	1075.27 ± 61.97		1.11 ± 0.064
20% W/O	117.78 ± 5.61		1.31 ± 0.063	1087.00 ± 51.83		1.12 ± 0.054

* Abbreviations: CAT, catalase; SOD, superoxide dismutase; GPX, glutathione peroxidase; GST, glutathione S-Transferase; QR, NAD(P)H: quinone oxidoreductase. UA/mg protein and induction rate (treated/control) were calculated.

**Table 2:** Protein tentative identification by LC-MS/MS in the 20% and 40% fractions obtained from crude homogenates of *A. sulcata* with and without symbiont.

Family	UniProt Accession Number	Protein			Organism	Mass (Da)	Score			
		Recommended Name	Short Name	Alternative Name			20% W	20% W/O	40% W	40% W/O
Sea anemone sodium channel (Nav) inhibitory toxin family. Type I subfamily	P0DL49	Delta-actitoxin-Avd1c 1	Delta-AITX-Avd1c 1	A. viridis toxin 2 (Av2; Avt2) Neurotoxin 2 Toxin 2c1; Toxin 2c4; Toxin Av2-1	*A. viridis*	8999	21 ± 0	-	-	265 ± 38
	B1NWT7	Delta-actitoxin-Avd1c 4	Delta-AITX-Avd1c 4	Toxin 2c2 Toxin 2c3	*A. viridis*	8870	-	-	-	238
	P01529	Delta-actitoxin-Avd1d	Delta-AITX-Avd1d	Neurotoxin 5 ATX-V; As5; Toxin V	*A. sulcata*	5222	-	-	1017 ± 210	278
	P0DL52	Delta-actitoxin-Avd1e 1	Delta-AITX-Avd1e 1	Neurotoxin 2; Av2 Toxin 2-4; Toxin 2c5	*A. viridis*	9013	-	-	1253 ± 179	264 ± 50
	P0DMZ1	Delta-actitoxin-Avd1h	Delta-AITX-Avd1h	Neurotoxin 1-1	*A. viridis*	9116	-	-	83 ± 24	-
Sea anemone sodium channel (Nav) inhibitory toxin family	A0A0S1M162	Sodium channel toxin protein, partial	-	-	*A. sulcata*	9234	-	-	291 ± 71	278 ± 27
Sea anemone type 1 potassium channel (Kv1) toxin family. Type 1b subfamily	Q9TWG1	Kappa-actitoxin-Avd6a	Kappa-AITX-Avd6a	Kaliseptine (AsKS)	*A. sulcata*	4180	26 ± 5	53 ± 9	-	-
	P0DN00	U-actitoxin-Avd9a	U-AITX-Avd9a	Potassium channel toxin Avtx-6	*A. viridis*	9383	-	-	-	23
Sea anemone type 1 potassium channel (Kv1) toxin family	A0A0S1M179	Type I potassium channel toxin protein	-	-	*A. sulcata*	7957	-	-	73 ± 28	65 ± 40
Venom Kunitz-type family. Sea anemone type 2 potassium channel (Kv2) toxin subfamily	Q9TWG0	KappaPI-actitoxin-Avd3b	KappaPI-AITX-Avd3b	Kunitz-type serine protease inhibitor Kalicludine-1 (AsKC1)	*A. sulcata*	7028	639 ± 51	1248 ± 48	784 ± 33	997 ± 166
	Q9TWF9	KappaPI-actitoxin-Avd3c	KappaPI-AITX-Avd3c	Kalicludine-2 (AsKC2)	*A. sulcata*	7115	545 ± 59	1009 ± 68	725	700
	Q9TWF8	KappaPI-actitoxin-Avd3d	KappaPI-AITX-Avd3d	Kalicludine-3 (AsKC3)	*A. sulcata*	7075	440 ± 91	418 ± 3	524 ± 26	478 ± 89
	P0DN07	U-actitoxin-Avd3g	U-AITX-Avd3g	AsKC4	*A. viridis*	9709	294 ± 38	415 ± 32	386 ± 51	414 ± 77
	P0DN13	U-actitoxin-Avd3l	U-AITX-Avd3l	AsKC9	*A. viridis*	9813	278 ± 12	435 ± 36	443 ± 65	438 ± 106
	P0DN15	U-actitoxin-Avd3n	U-AITX-Avd3n	AsKC11	*A. viridis*	10,398	-	-	307	-
	P0DN16	U-actitoxin-Avd3o	U-AITX-Avd3o	AsKC12	*A. viridis*	10,415	-	-	310 ± 48	-
Sea anemone type 3 (BDS) potassium channel (Kv3) toxin family	P0DMX7	Kappa-actitoxin-Avd4c	Kappa-AITX-Avd4c	Blood depressing substance 3 (BDS-3)	*A. viridis*	8895	486	-	1778 ± 312	2116
	P0DMX8	Kappa-actitoxin-Avd4d	Kappa-AITX-Avd4d	Blood depressing substance 4 (BDS-4)	*A. viridis*	8939	200	-	1963 ± 408	2164 ± 165
	P0DMX9	Kappa-actitoxin-Avd4e	Kappa-AITX-Avd4e	Blood depressing substance 5 (BDS-5)	*A. viridis*	8827	215	621 ± 28	-	1843 ± 135
	P0DMY1	Kappa-actitoxin-Avd4g	Kappa-AITX-Avd4g	Blood depressing substance 7 (BDS-7)	*A. viridis*	8807	-	-	840 ± 101	-
	P0DMY4	Kappa-actitoxin-Avd4j	Kappa-AITX-Avd4j	Blood depressing substance 10 (BDS-10)	*A. viridis*	9492	-	-	482 ± 44	625
	P0DMY7	Kappa-actitoxin-Avd4m	Kappa-AITX-Avd4m	Blood depressing substance 13 (BDS-13)	*A. viridis*	8845	121 ± 41	20	727 ± 46	810 ± 19
	A0A0S1M190	Type III potassium channel toxin protein, partial	-	-	*A. sulcata*	8923	-	-	1767 ± 311	1947 ± 202
	A0A0S1M170	Type III potassium channel toxin protein, partial	-	-	*A. sulcata*	9520	-	-	481 ± 44	662 ± 34
	A0A0S1M169	Type III potassium channel toxin protein, partial	-	-	*A. sulcata*	9195	-	-	200 ± 28	1058 ± 118
	A0A0S1M1A6	Type III potassium channel toxin protein	-	-	*A. sulcata*	9539	-	-	214 ± 3	466 ± 170
	A0A0S1M174	Type III potassium channel toxin protein, partial	-	-	*A. sulcata*	9436	-	-	66 ± 17	50 ± 14
	A0A0S1M194	Type III potassium channel toxin protein	-	-	*A. sulcata*	7882	-	-	-	67 ± 25
	A0A0S1M195	Type III potassium channel toxin protein	-	-	*A. sulcata*	8327	-	-	-	36
	A0A0S1M185	Type III potassium channel toxin protein	-	-	*A. sulcata*	8284	-	-	26	-
Non-classical Kazal-type elastase inhibitor	P16895	PI-actitoxin-Avd5a	PI-AITX-Avd5a	Non-classical Kazal-type elastase inhibitor	*A. sulcata*	5472	-	-	60	27
Sea anemone 8 toxin family	P0DMZ3	U-actitoxin-Avd8a	U-AITX-Avd8a	Avtx-1	*A. viridis*	9128	-	-	521 ± 46	530 ± 46
	P0DMZ6	U-actitoxin-Avd8d	U-AITX-Avd8d	Avtx-4	*A. viridis*	9527	47 ± 24	50 ± 12	155 ± 35	174 ± 6
	P0DMZ7	U-actitoxin-Avd8e	U-AITX-Avd8e	Avtx-5	*A. viridis*	9874	-	-	37 ± 3	85
Sea anemone structural class 9a family	P0DMZ8	U-actitoxin-Avd13a/b Cleaved into 2 chains: Avd13a and Avd13b	U-AITX-Avd13a/b	Peptide toxin AV-1	*A. viridis*	18,716	71	195 ± 38	-	149 ± 77
Small cysteine-rich protein (SCRiP) family	P0DL61	Small cysteine-rich protein (Fragment)	Avir_SCRiP; SCRiP	-	*A. viridis*	7717	35 ± 5	-	21	71 ± 2
**Family**	**UniProt Accession Number**	**Protein**			**Organism**	**Mass (Da)**	**Score**			
		**Recommended name**	**Short name**	**Alternative name**			**20% W**	**20% W/O**	**40% W**	**40% W/O**
Antioxidant enzymes	Q8I807	Copper/zinc superoxide dismutase (CuZnSODb)			*A. viridis*	15,950	131 ± 35	108 ± 6	924 ± 117	526 ± 9
	A0EJ86	Catalase			*A. viridis*	57,729	30 ± 5	112 ± 29	483 ± 105	580 ± 164
	A0A1D8RF20	Glutathione peroxidase			*A. viridis*	28,042	-	31 ± 7	163 ± 69	203 ± 75
	A0A1D8RF24	Glutathione peroxidase			*A. viridis*	18,957	-	-	42 ± 23	26
	Q9GPI6	Green fluorescent protein as(S)FP499—Chain A			*A. sulcata*	25,401	390 ± 78	1224 ± 50	3282 ± 242	2547 ± 141
	Q9GZ28	GFP-like non-fluorescent chromoprotein FP595—Cleaved into 2 chains: Chain 1 and chain 2			*A. sulcata*	26,301	333 ± 99	1018 ± 44	2340 ± 498	2213 ± 210

## Data Availability

Not applicable.

## Supplementary Materials

The following supporting information can be downloaded at: https://www.mdpi.com/article/10.3390/ijms241411249/s1.
