# Supplementary material for: Antiproliferative, Antioxidant, Chemopreventive and Antiangiogenic Potential of Chromatographic Fractions from Anemonia sulcata with and without Its Symbiont Symbiodinium in Colorectal Cancer Therapy
Source: Int J Mol Sci. 2023 Jul 8;24(14):11249. doi: 10.3390/ijms241411249 (PMC10379856; doi:10.3390/ijms241411249)
Supplement: Supplementary file 1 [file ijms-24-11249-s001.zip › ijms-2475808-supplementary.pdf]

Antiproliferative, antioxidant, chemopreventive and antiangiogenic potential of chromatographic fractions from *Anemonia sulcata* with and without its symbiont *Symbiodinium* in colorectal cancer therapy

Mercedes Peña [1,2,3], Cristina Mesas [1,2,3], Gloria Perazzoli [1,2,3], Rosario Martínez [4], Jesús M Porres [4], Kevin Doello [1,3,5], Jose Prados [1,2,3,*], Consolación Melguizo [1,2,3,†] and Laura Cabeza [1,2,3,†]

1 Institute of Biopathology and Regenerative Medicine (IBIMER), Center of Biomedical Research (CIBM), University of Granada, 18100 Granada, Spain; mpenacontreras@ugr.es (M.P.); cristinam@correo.ugr.es (C.M.); gperazzoli@ugr.es (G.P.); kdoello@correo.ugr.es (K.D.); melguizo@ugr.es (C.M.); lautea@ugr.es (L.C.)
2 Department of Anatomy and Embryology, Faculty of Medicine, University of Granada, 18071 Granada, Spain
3 Biosanitary Institute of Granada (ibs.GRANADA), SAS-University of Granada, 18014 Granada, Spain
4 Department of Physiology, Institute of Nutrition and Food Technology (INyTA), Center of Biomedical Research (CIBM), University of Granada, 18100 Granada, Spain; rosariomz@ugr.es (R.M.); jmporres@ugr.es (J.M.P.)
5 Medical Oncology Service, Virgen de las Nieves Hospital, 18016 Granada, Spain
* Correspondence: jcprados@ugr.es
† These authors contributed equally to this work.

Supplementary material

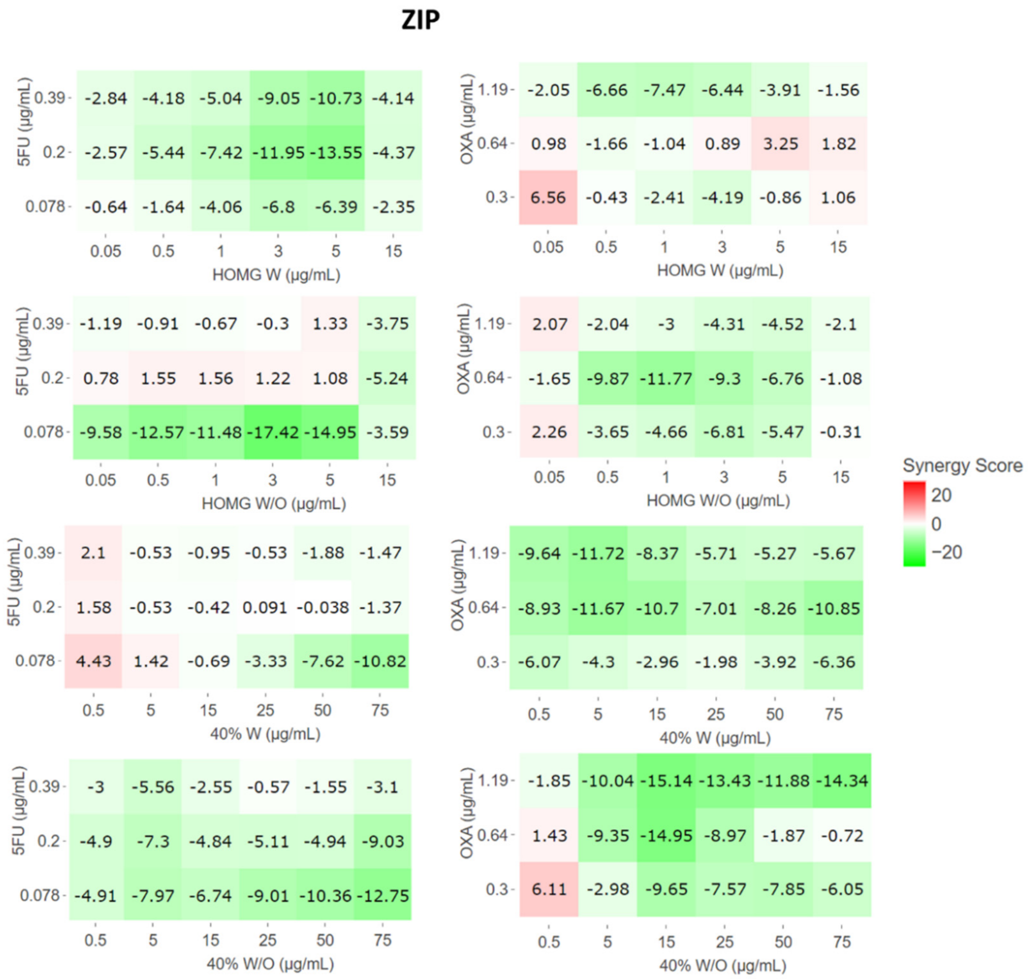

Figure S1. Synergistic effect of HOMG or 40% fraction from *A. sulcata* W and W/O symbiont, in combination with (A) 5FU or (B) OXA by ZIP model. Different concentrations of HOMG W, W/O, or 40% W, W/O were co-incubated with 5FU (0.08, 0.2 and 0.39 µg/mL) or OXA (0.3, 0.64 and 1.19 µg/mL) for 72 hours. Synergy scores (ZIP model) were represented in Heatmaps with SynergyFinder Plus, where synergy (values >10, red), additive effect (from -10 to 10, white) and antagonism (< -10, green) areas were distinguished.

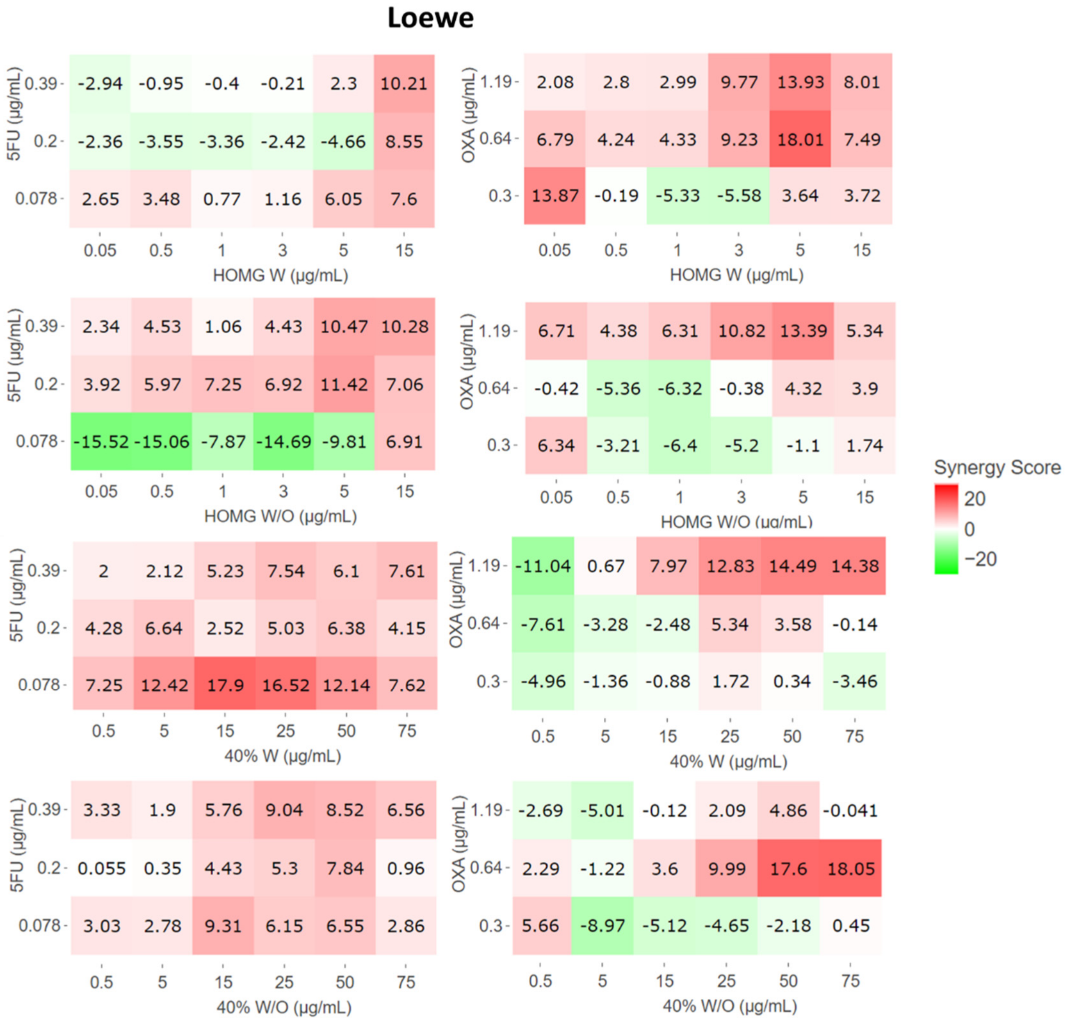

Figure S2. Synergistic effect of HOMG or 40% fraction from *A. sulcata* W and W/O symbiont, in combination with (A) 5FU or (B) OXA by Loewe model. Different concentrations of HOMG W, W/O, or 40% W, W/O were co-incubated with 5FU (0.08, 0.2 and 0.39 µg/mL) or OXA (0.3, 0.64 and 1.19 µg/mL) for 72 hours. Synergy scores (Loewe model) were represented in Heatmaps with SynergyFinder Plus, where synergy (values >10, red), additive effect (from -10 to 10, white) and antagonism (< -10, green) areas were distinguished.

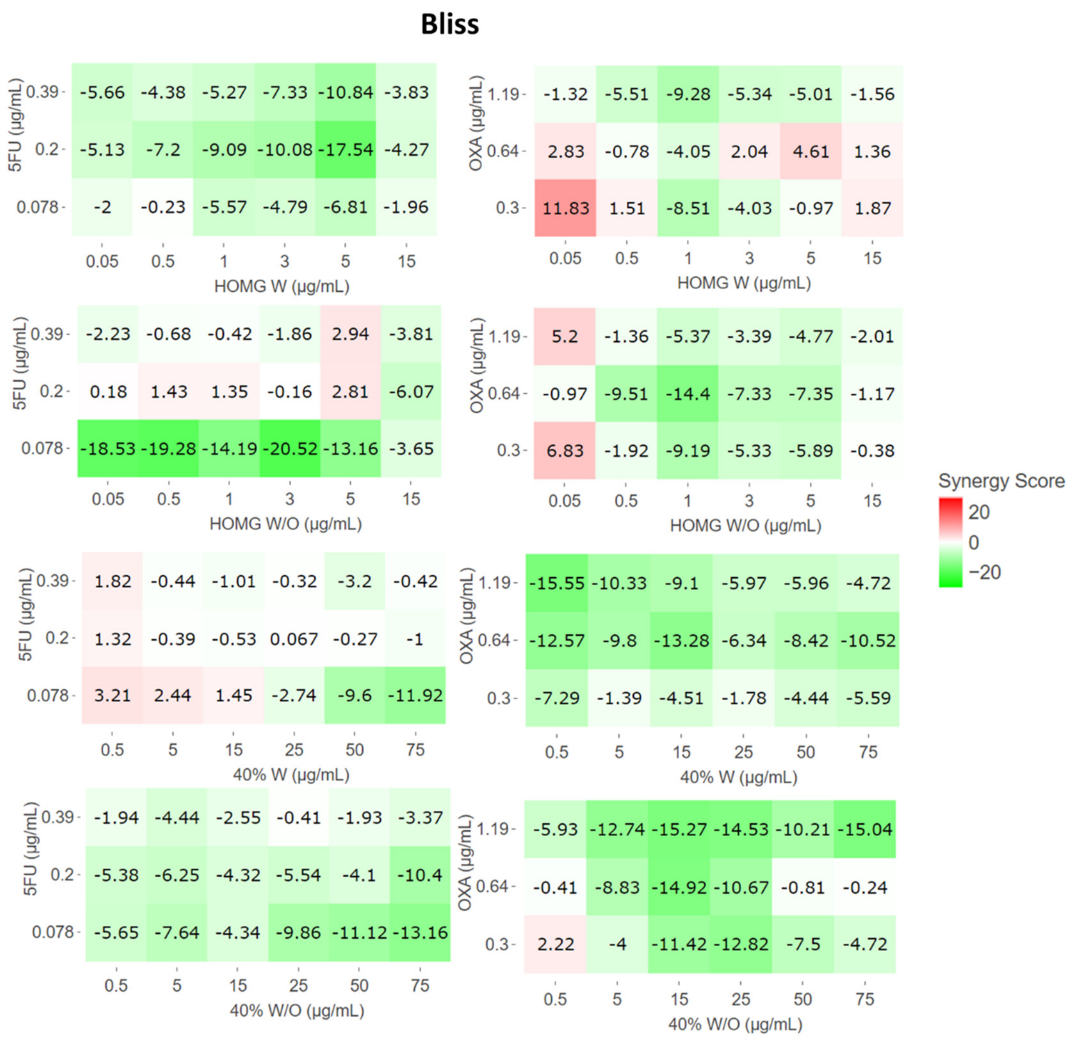

Figure S3. Synergistic effect of HOMG or 40% fraction from *A. sulcata* W and W/O symbiont, in combination with (A) 5FU or (B) OXA by Bliss model. Different concentrations of HOMG W, W/O, or 40% W, W/O were co-incubated with 5FU (0.08, 0.2 and 0.39 μg/mL) or OXA (0.3, 0.64 and 1.19 μg/mL) for 72 hours. Synergy scores (Bliss model) were represented in Heatmaps with SynergyFinder Plus, where synergy (values >10, red), additive effect (from -10 to 10, white) and antagonism (< -10, green) areas were distinguished.

Table S1. Major tentative identification of compounds in the 20% and 40% fractions of *Anemonia sulcata* W and W/O symbiont by HPLC-MS analysis.

Peak	Fraction	*RT	Adduct Ion **m/z*	Error	Suggested *MF (Calc. *MW)	Suggested Compounds	
						**Common Name**	**Category, Main Class**
**P1**	20% W	4.44	274.2742 [M+NH4]	1	C16H35NO2 (273.27)	C16 Sphinganine	SP, Sphingoid bases
	20% W/O	4.44	274.2740 [M+NH4]	1			
	40% W	4.44	274.2736 [M+NH4]	1			
	40% W/O	4.46	274.2736 [M+NH4]	2			
**P2**	20% W	5.65	304.3000 [M+H]	2	C21H37N (303.29)	-	-
	20% W/O	5.6	304.2994 [M+H]	2			
	40% W	5.59	304.2994 [M+H]	2			
	40% W/O	5.59	304.2995 [M+H]	1			
	20% W/O	5.74	482.3601 [M+H]	1	C24H52NO6P (481.35)	PC(O-16:0/0:0)	GP, PC
	40% W	5.64	482.3599 [M+H]	1			
	40% W/O	5.62	482.3598 [M+H]	1			
**P3**	20% W	6.5	331.2846 [M+H]	1	C19H38O4 (330.28)	MG(16:0/0:0/0:0)	GL, MG
	20% W/O	6.45	331.2838 [M+H]	2			
	40% W	6.41	331.2836 [M+H]	2			
	40% W/O	6.43	331.2838 [M+H]	2			
**P4**	20% W	7	359.3161 [M+H]	1	C21H42O4 (358.31)	MG(18:0/0:0/0:0)[rac]	GL, MG
	20% W/O	6.95	359.3153 [M+H]	1			
	40% W	6.92	359.3149 [M+H]	1			
	40% W/O	6.94	359.3150 [M+H]	1			
**P5**	20% W	7.36	643.5181 [M+H-H2O]	2	C36H73N2O6P (660.52)	CerPE(d14:1(4E)/20:0) CerPE(d16:1(4E)/18:0)	SP, PS
	20% W/O	7.26	643.5165 [M+H-H2O]	2			
	40% W/O	7.33	643.5159 [M+H-H2O]	2			
**P6**	20% W	8.56	381.3519 [M+H-H2O]	1	C28H46O (398.36)	Brassicasterol	ST
	20% W/O	8.59	381.3512 [M+H-H2O]	1			
**P7**	20% W	8.88	369.3519 [M+H-H2O]	1	C27H46O (386.36)	Cholesterol 19-Norergost-5-en-3-ol; (3β,24ξ)-form	ST
	20% W/O	8.89	369.3512 [M+H-H2O]	1			
	40% W	8.95	369.3507 [M+H-H2O]	3			
	20% W/O	8.93	536.5032 [M+H]	1	C34H65NO3 (535.5)	Cer(d18:2/16:0); Cer(d14:1/20:1)	SP, Cer
**P8**	20% W/O	4.78	500.2767 [M+H]	1	C25H42NO7P (499.27)	PE(20:5(5Z,8Z,11Z,14Z,17Z)/0:0)	GP, PE
**P9**	20% W/O	5	466.2923 [M+H]	1	C22H44NO7P (465.29)	PC(14:1(9Z)/0:0); PE(17:1(9Z)/0:0)	GP, PC/PE
**P10**	20% W/O	8.2	657.5320 [M+H-H2O]	2	C37H75N2O6P (674.54)	PE-Cer(d14:1(4E)/21:0); PE-Cer(d15:1(4E)/20:0); PE-Cer(d16:1(4E)/19:0)	SP, PS
**P11**	40% W	3.61	236.2005 [M+Na]	9	C13H27NO (213.21)	Tridecanamide	FA, Fatty amides
**P12**	40% W	4.85	415.2110 [M+H]	3	C17H30N6O4S (414.20)	His Lys Met	Tripeptide
			432.2373 [M+H]	4	C20H29N7O4 (431.23)	Arg Trp Ala	Tripeptide

**P13**	40% W	5.35	496.3392 [M+H]	1	C24H50NO7P (495.33)	PC(16:0/0:0); PE(19:0/0:0)	GP, PC/PE
	40% W/O	5.34	496.3389 [M+H]	2			
**P14**	40% W	6.08	332.3305 [M+NH4]	2	C23H38 (314.30)	3Z,6Z,9Z,12Z,15Z-Tricosapentaene	FA, Hydrocarbons
	40% W/O	6.08	332.3307 [M+NH4]	2			
**P15**	40% W	7.82	460.2141 [M+H]	6	C20H31N5O6 (437.23)	Lys Gln Tyr	Tripeptide
	40% W/O	7.88	460.2142 [M+H]	6			
**P16**	40% W	5.01	302.3047 [M+H]	2	C18H39NO2 (301.30)	Sphinganine	SP, Sphingoid bases
	40% W/O	5.01	302.3049 [M+H]	2			
**P17**	40% W	8.2	338.3411 [M+H]	2	C22H43NO (337.33)	Erucamide	FA, Fatty amides
	40% W/O	8.24	338.3411 [M+H]	2			

* Abbreviations: RT (Retention time, min); m/z (mass/charge number of ions); MF (Molecular formule); Calc. MW (Calculated Molecular Weight). Cer (ceramides); FA (fatty acids/acyls); GL (glycerolipids); GP (glycerophospholipids); MG (monoradylglycerols); PC (phosphatidylcholine); PE (phosphatidylethanolamine); PS (Phosphosphingolipids); SP (sphingolipids); ST (sterols).

Table S2. Comparison of the amino acid sequences of each of the BDS toxins identified by LC-MS/MS. Sequences have been extracted from Uniprot database. Sequence differences are marked in red and the RGD motif is highlighted in blue.

BDS toxin	Amino acid sequences
**BDS-3**	MNKALFLCLVVLCAAVVFAAEDLQKAKHAPFKRAAPCFCPGKPDRGDLWIFRGTCPGGYGYTSNCYKWPNICCYPH
**BDS-4**	MNKALFLCLVVLCAAVVFAAEDLQKAKHVPFKRAAICFCPGKPDRGDLWIFRGTCPGGYGYTSNCYKWPNICCYPH
**BDS-5**	MNKALFLCLVVLCAAVVFAAEDLQKAKHAPFKRAAPCFCSGKPGRGDLWIFRGTCPGGYGYTSNCYKWPNICCYPH
**BDS-7**	MNKALFLCLVVLCAAVVFAAEDLQKAKHAPFKRAAPCFCSGKPGRGDLWIL-RGDCPGGYGYTSNCYKWPNICCYPH
**BDS-10**	MNKALFLCLVVLCAAVVFAAEDLQKAKHAPFKRGAQVCFCPGKVDRGDLWIL-RGDCPGGYGYTSNCYTWPNICCYPQSFSGR
**BDS-13**	MNKALFLCLVVLCAAVVFAAEDLQKAKHAPFKRATACFCPGKADRGDLWIL-RGDCPDGYGYTTYCYKGPNICCYPH

Table S3. List of primers used in the RT-qPCR experiments.

Gene	Accession number	Primer		Tm°
		**Sense**	**Sequence**	
GAPDH	P04406	Forward	TCGGAGTCAACGGATTTG	62.4
		Reverse	CAACAATATCCACTTTACCAGAG	59.1
CD24	P25063	Forward	CAGTAGTCTTGATGACCAAAG	56.4
		Reverse	ACAGCATTCTGGAATAAAGC	58.4
CD44	P16070	Forward	TTATCAGGAGACCAAGACAC	56.6
		Reverse	ATCAGCCATTCTGGAATTTG	61.5
CD133	O43490	Forward	AAGCATTGGCATCTTCTATG	59.3
		Reverse	TTTGCTCTGGAGTTTCATTC	59.4
Oct-4	Q01860	Forward	GATCACCCTGGATATACAC	58.1
		Reverse	GCTTTGCATATCTCCTGAAG	59.1
SOX2	P48431	Forward	ATAATAACAATCATCGGCGG	61.1
		Reverse	AAAAAGAGAGAGGCAAACTG	57.8
NANOG	Q9H9S0	Forward	CCAGAACCAGAGAATGAAATC	60.1
		Reverse	TGGTGGTAGGAAGAGTAAAG	55.9
